# Follow‐up frequency and clinical outcomes in patients with type 2 diabetes: A prospective analysis based on multicenter real‐world data

**DOI:** 10.1111/1753-0407.13271

**Published:** 2022-05-25

**Authors:** Qiubo Zhao, Hongwei Li, Qicheng Ni, Yuancheng Dai, Qidong Zheng, Yufan Wang, Tingyu Ke, Li Li, Dong Zhao, Qijuan Dong, Bangqun Ji, Juan Shi, Ying Peng, Yifei Zhang, Fengmei Xu, Weiqing Wang

**Affiliations:** ^1^ Department of Endocrinology and Metabolism, Hebi Coal (group) Ltd General Hospital Hebi China; ^2^ Department of Endocrine and Metabolic Diseases, Shanghai Institute of Endocrine and Metabolic Diseases, Ruijin Hospital Shanghai Jiao Tong University School of Medicine Shanghai China; ^3^ Shanghai National Clinical Research Center for metabolic Diseases, Key Laboratory for Endocrine and Metabolic Diseases of the National Health Commission of the PR China, Shanghai Key Laboratory for Endocrine Tumor, State Key Laboratory of Medical Genomics, Ruijin Hospital Shanghai Jiao Tong University School of Medicine Shanghai China; ^4^ Department of Internal Medicine of Traditional Chinese Medicine Sheyang Diabetes Hospital Yancheng China; ^5^ Department of Internal Medicine The Second People's Hospital of Yuhuan Yuhuan China; ^6^ Department of Endocrinology and Metabolism, Shanghai General Hospital Shanghai Jiao Tong University School of Medicine Shanghai China; ^7^ Department of Endocrinology The Second Affiliated Hospital of Kunming Medical University Kunming China; ^8^ Department of Endocrinology Ningbo First Hospital China; ^9^ Center for Endocrine Metabolism and Immune Diseases, Beijing Luhe Hospital Capital Medical University Beijing China; ^10^ Department of Endocrinology and Metabolism People's Hospital of Zhengzhou Affiliated Henan University of Chinese Medicine Zhengzhou China; ^11^ Department of Endocrinology Xingyi People's Hospital Xingyi China

**Keywords:** cost‐effectiveness, follow‐up frequency, glycemic control, type 2 diabetes mellitus, 2型糖尿病, 随访频率, 血糖控制, 成本效益

## Abstract

**Background:**

To determine whether the follow‐up frequency for type 2 diabetes mellitus (T2DM) patients in the National Metabolic Management Centers (MMCs) leads to different clinical outcomes.

**Methods:**

A total of 19 908 T2DM patients with at least 6 months of facility‐based follow‐up were recruited in MMCs between June 2017 and April 2021 and divided into lower‐frequency and higher‐frequency follow‐up (LFF and HFF) groups according to the median follow‐up frequency of 2.0 (interquartile range 1.2) times per year. Metabolic parameters at baseline and at the last follow‐up visit were analyzed. Multivariable linear regression models were performed to assess the relationship between follow‐up frequency and between‐group percentage changes, adjusting for the major covariables. Additional stratified analyses were conducted to evaluate the metabolic outcomes in the subgroups.

**Results:**

The characteristics of the participants in the LFF and HFF groups were significantly different at baseline. Participants had significant improvements in multiple metabolic parameters after follow‐up. Patients with HFF showed significantly greater decrease in percentage changes of fasting blood glucose (−4.95% ± 37.96% vs −2.21% ± 43.08%, *P* < .0001) and glycosylated hemoglobin (HbA1c) (−12.14% ± 19.78% vs −9.67% ± 20.29%, *P* < .0001) after adjustments compared to those with LFF. Furthermore, stratification analyses showed that significant between‐group percentage changes of HbA1c were observed in those with younger age (<55 years) and higher HbA1c (>9%) at baseline (*P* for interaction <.001).

**Conclusions:**

HFF is associated with better metabolic outcomes. Participants, especially with younger age or worse HbA1c at baseline in the HFF group achieved better glycemic control than those in the LFF group.

## Graphical abstract

**Highlights**

- In this prospective analysis based on multicenter real‐world data, higher‐frequency follow‐up achieved better glycemic control compared with lower‐frequency follow‐up in patients with type 2 diabetes mellitus (T2DM).
- Furthermore, stratification analysis indicated that metabolic benefits with higher‐frequency follow‐up were predominant in patients with younger age or worse glycosylated hemoglobin. This study provides evidence‐based recommendations for more efficient follow‐up policies for targeted T2DM patients with different baseline characteristics.

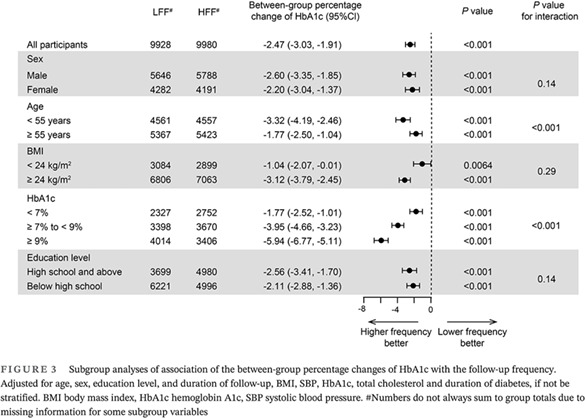

## 摘要

**背景:**

本研究旨在确定国家代谢管理中心(MMC)对2型糖尿病(T2D)患者的随访频率是否会导致不同的临床结果。

**方法:**

选择2017年6月至2021年4月在MMC进行至少6个月随访的2型糖尿病患者19,908例, 根据每年2.0次(IQR, 1.2)的中位数随访频率, 分为低频组(LFF)和高频组(HFF)。分析基线和末次随访时的代谢参数。采用多变量线性回归模型评估经主要协变量校正后的随访率和组间百分比变化之间的关系。另外还进行了分层分析, 以评估各亚组的代谢结果。

**结果:**

LFF组和HFF组的受试者在基线时的特征有显著差异。随访后, 受试者的多项代谢参数均有显著改善。校正后HFF组的空腹血糖(‐4.95±37.96% vs ‐2.21±43.08%, P<0.0001)和糖化血红蛋白(HbA1c) (‐12.14±19.78% vs ‐9.67±20.29%, P<0.0001)比LFF组显著下降。此外, 分层分析显示, 在基线时年龄较小(<55岁)和HbA1c较高(>9%)的人群中, HbA1c的组间百分比变化显著(P<0.001)。

**结论:**

HFF与较好的代谢结局有关。HFF组较年轻或基线HbA1c较差的受试者, 其血糖控制效果好于LFF组。

## 1 INTRODUCTION

Diabetes is the fastest increasing disease worldwide and has become a major public health issue in China.^1^, ^2^ Type 2 diabetes mellitus (T2DM) involves multiple comorbid conditions that require effective lifelong care and continuous management.^3^ The burden of diabetes has increased faster in low‐income and middle‐income countries than in high‐income countries because of increasing prevalence and financial costs.^4^ The National Metabolic Management Centers (MMCs) are an innovative project for the management of metabolic diseases and complications throughout China.^5^ With the big database of the MMCs, real‐world studies are becoming a powerful tool to understand the impact of current practices on clinical courses and outcomes, such as screening for diabetic retinopathy^6^ and development of arterial stiffness,^7^ as previously reported by our group. Regular monitoring is necessary and important to keep T2DM under control. The increasing diabetes population has resulted in increased costs and overburdened physicians.^8^ Developing an efficient model of diabetes care is essential to manage the overwhelming number of T2DM patients. Identifying productive follow‐up frequency for managing chronic diabetes will reduce the population‐level economic and health care burden from diabetes. However, there are large variations in the frequency of follow‐up across different regions, and evidence‐based recommendations are lacking.^9^ Several studies^10^, ^11^, ^12^, ^13^, ^14^, ^15^ have investigated the relationship between follow‐up frequency and metabolic outcomes in patients with T2DM. Indeed, because of the different study designs, conclusions led to controversies.^10^, ^11^, ^12^, ^13^, ^14^, ^15^ These observations prompted us to further investigate the association of follow‐up frequency and glycemic control. It is important to provide real‐world outcomes for follow‐up frequency associated with achieving metabolic benefits at lower cost. Here, we provide a prospective analysis based on multicenter, real‐world data from a large population on the frequency of follow‐up in a facility‐based Chinese T2DM cohort in MMCs. In our study, we divided 19 908 T2DM patients into lower‐frequency and higher‐frequency follow‐up (LFF and HFF) groups (according to the median follow‐up frequency) and evaluated and compared the metabolic parameters at baseline and at the last follow‐up visit. This study provides additional evidence for the association between follow‐up frequency and clinical advantages for T2DM patients.

## 2 METHODS

### 2.1 Cohort description

In this prospective, observational, real‐world study, a database of 23 415 adult participants with T2DM who had at least one follow‐up visit was recruited from 10 MMCs between June 2017 and April 2021. T2DM was identified according to the WHO criteria.^16^ A detailed introduction of the MMC program can be found in previous publications (ClinicalTrials.gov number NCT03811470).^5^, ^6^, ^7^, ^17^, ^18^ Briefly, the MMCs are an innovative project for the management of diabetes and other metabolic diseases throughout China, with “one center, one stop, and one standard” as its core principle. The MMCs have implemented a series of changes to integrate the advanced medical equipment and internet of things into the system and aimed at providing patients with highly efficient diagnosis and care both in and out of hospital.

**FIGURE 1:**
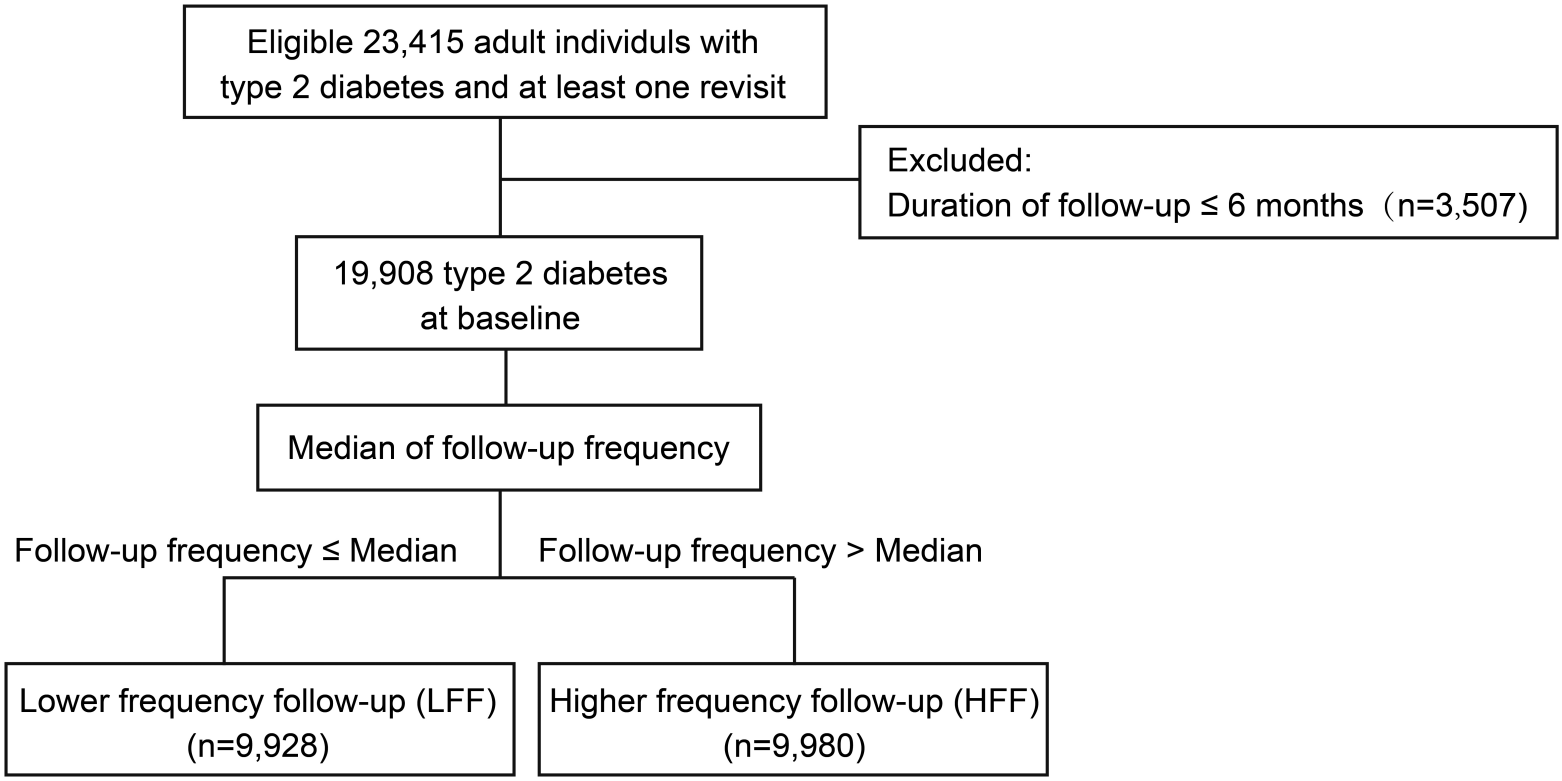
Flowchart of the study

Participants with a follow‐up duration ≤6 months (n = 3507) were excluded, and finally 19 908 participants were included for the main analysis (Figure 1). At the time of recruitment, all data were collected in local MMCs by trained staff according to a standard protocol.^5^, ^6^, ^7^ Education level was categorized as lower than high school or high school and above. After the baseline survey, participants were advised to have regular follow‐ups at MMCs. Individualized treatment goals were set for the T2DM patients at each MMC at the beginning of enrolment into the MMC program based on their characteristics. The MMC system was explained to the patients as a convenient mode for regular revisits. The guideline for the prevention and treatment of T2DM in China was strictly followed to carry out standardized and comprehensive management for T2DM patients. Currently, the patient follow‐up frequency recommended by the MMC‐related standard operation procedure is two to four visits per year, and the actual frequency can be adjusted by the physicians themselves according to the patients' metabolic status and other situations, including personal propensity. MMCs provide internet‐based self‐management support (app, social software platform, etc) for patients, including health education information and courses, blood glucose reporting and tracking, online lectures, and Q&As provided by the doctors in the MMCs.

We divided all the eligible participants into two groups according to the median follow‐up frequency: below and equal to the median follow‐up frequency was defined as LFF and above the median of follow‐up frequency was defined as HFF.

### 2.2 Statistical analysis

Continuous variables were mean ± SD or median (interquartile range) values. Categorical variables were summarized as group numbers (n%). The demographic and clinical characteristics were compared with the chi‐square test for categorical variables and with one‐way analysis of variance for continuous variables. The comparisons of continuous variables were performed using the paired sample *t* test between baseline and follow‐up in the LFF and HFF groups. We constructed a between‐group comparison using multivariable linear regression models to assess the relationship between follow‐up frequency and between‐group percentage changes adjusting for major covariables including age, sex, education level, duration of follow‐up, body mass index (BMI), systolic blood pressure (SBP), glycosylated hemoglobin (HbA1c), total cholesterol, and duration of diabetes. In addition, a stratified analysis of the association between the percentage changes in HbA1c between groups and follow‐up frequency was performed using the interaction test. Results were adjusted for major covariables including age, sex, education level, duration of follow‐up, BMI, SBP, HbA1c, total cholesterol, and duration of diabetes, unless stratified. *P* < .05 was considered to be statistically significant. All statistical analyses were performed using R statistics (version 4.0.5).

## 3 RESULTS

### 3.1 Characteristics of participants

In total, 19 908 participants with diabetes from 10 nationwide MMCs were enrolled in the final analysis (Figure 1). The general characteristics of the study participants are presented in Table 1. The mean (SD) age of the study population was 54.6 (11.0) years old, and 11 434 (57.4%) were men (Table 1). The mean (SD) follow‐up duration was 20.1 (9.6) months (Table 1).

**TABLE 1:** Baseline characteristics of T2DM participants within MMCs

	Total	LFF	HFF	*P* value
n	19 908	9928	9980	
Age (y)	54.6 ± 11.0	54.9 ± 10.6	54.3 ± 11.4	<.0001
Male, n (%)	11 434 (57.4%)	5646 (56.9%)	5788 (58.0%)	.11
Duration of diabetes (y)	5.6 (1.2, 11.3)	5.8 (1.4, 11.3)	5.3 (0.9, 11.3)	.052
History of hypertension	8615 (43.4%)	4213 (42.6%)	4402 (44.3%)	.018
Education level high school and above, n (%)	8679 (43.6%)	3699 (37.3%)	4980 (50.0%)	<.0001
Ideal smoking, n (%)	14 805 (74.9%)	7338 (74.5%)	7467 (75.3%)	.19
Drinking, n (%)	2165 (10.9%)	1094 (11.1%)	1071 (10.8%)	.52
Fasting glucose (mmol/L)	9.34 ± 3.69	9.53 ± 3.92	9.16 ± 3.44	<.0001
Fasting C‐peptide (μg/L)	2.06 (1.40, 2.87)	2.07 (1.40, 2.89)	2.06 (1.41, 2.84)	.56
BMI (kg/m^2^)	26.0 ± 3.7	25.9 ± 3.7	26.1 ± 3.7	<.0001
Visceral fat area (cm^2^)	101.1 ± 41.0	99.8 ± 41.2	102.4 ± 40.6	<.0001
Waist circumference (cm)	91.7 ± 9.9	91.3 ± 9.9	92.1 ± 9.8	<.0001
SBP (mm Hg)	132.2 ± 18.6	132.4 ± 19.0	131.9 ± 18.2	.070
DBP (mm Hg)	77.4 ± 11.4	77.6 ± 11.4	77.1 ± 11.4	.001
HbA1c (%)	8.58 ± 2.09	8.74 ± 2.14	8.43 ± 2.03	<.0001
Triglyceride (mmol/L)	1.61 (1.11, 2.45)	1.62 (1.12, 2.50)	1.60 (1.10, 2.40)	.005
Total cholesterol (mmol/L)	4.90 ± 1.30	4.93 ± 1.28	4.88 ± 1.31	.009
HDL cholesterol (mmol/L)	1.20 ± 0.34	1.21 ± 0.34	1.19 ± 0.33	.004
LDL cholesterol (mmol/L)	2.95 ± 0.99	2.96 ± 0.98	2.94 ± 1.00	.13
Duration of follow‐up (mo)	20.1 ± 9.6	18.8 ± 8.9	21.3 ± 9.9	<.0001
HbA1c < 7%, n (%)	5079 (26.0%)	2327 (24.0%)	2752 (28.0%)	<.0001

*Note*: Data are presented as mean ± SD, median (25%, 75%), or n (%). The groups were compared via analysis of variance for the continuous outcomes and χ^2^ test for dichotomous variables (sex, education level, history of hypertension, ideal smoking, drinking, and HbA1c < 7%).

Abbreviations: BMI, body mass index; DBP, diastolic blood pressure; HbA1c, glycosylated hemoglobin; HDL, high‐density lipoprotein; HFF, higher‐frequency follow‐up; LDL, low‐density lipoprotein; LFF, lower‐frequency follow‐up; MMC, Metabolic Management Center; SBP, systolic blood pressure; T2DM, type 2 diabetes mellitus.

### 3.2 Participants' baseline characteristics associated with follow‐up frequency

**FIGURE 2:**
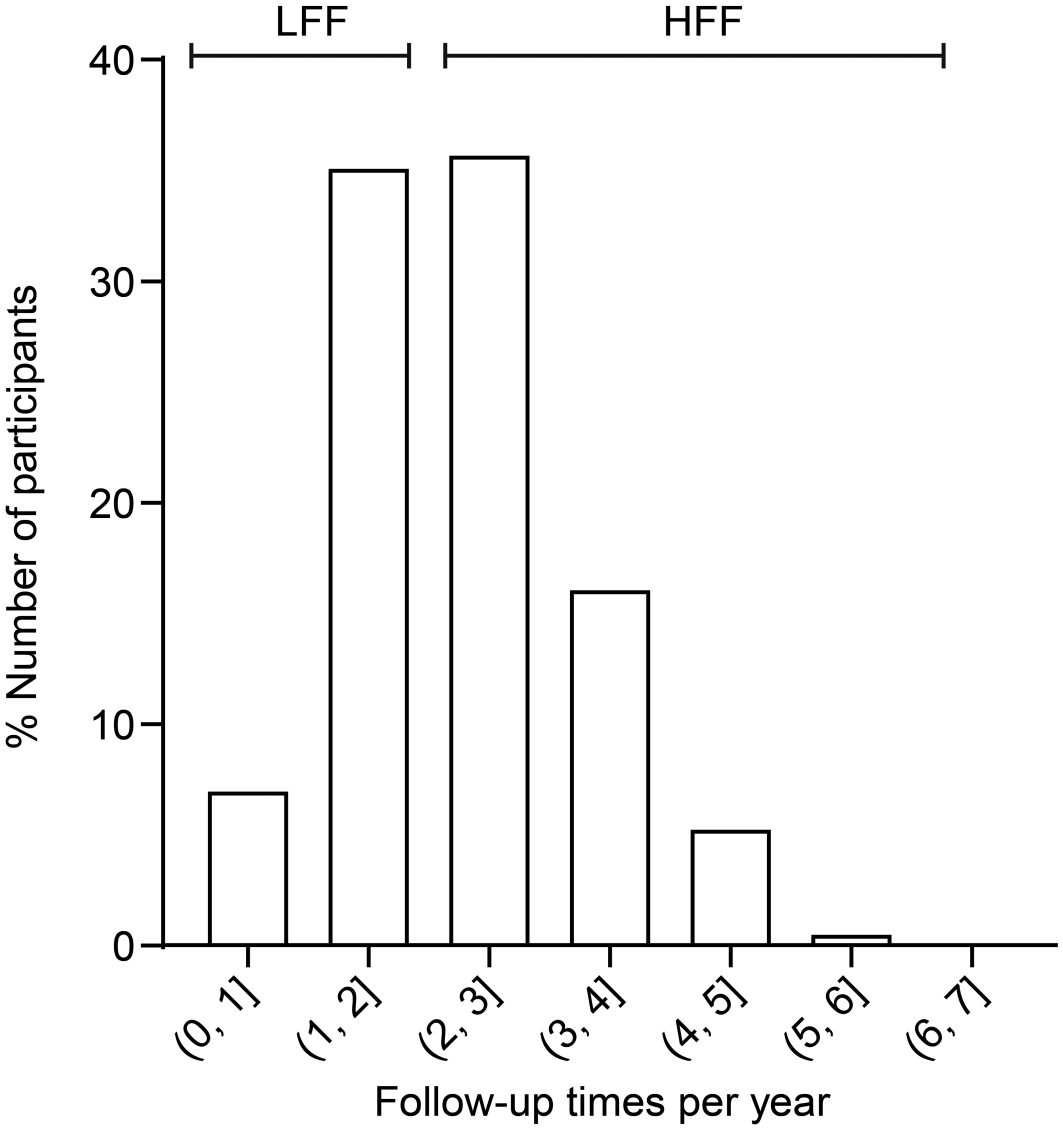
The histogram showing distribution of follow‐up times per year of 19 908 diabetic participants

The follow‐up frequencies for all the participants per year are shown in Figure 2. We divided all T2DM participants into LFF and HFF groups according to the median follow‐up frequency (2.0 times per year). The medians of the LFF and HFF groups were 1.7 and 2.9 times, respectively, per year. The metabolic outcomes at baseline and at the last visit were obtained and analyzed. At baseline, compared to those with LFF, patients with HFF were younger with higher education level, more likely to have a history of hypertension, and had lower levels of fasting blood glucose (FBG), HbA1c, total cholesterol, triglycerides, high‐density lipoprotein cholesterol, and diastolic blood pressure (DBP), but higher levels of BMI, visceral fat area, and waist circumference (all *P* values <.05, Table 1). The between‐group differences in sex, ideal smoking, drinking status, and duration of diabetes were not statistically significant (Table 1).

### 3.3 Percentage changes from baseline in multiple metabolic parameters with lower or higher follow‐up frequency

We analyzed the percentage changes of metabolic outcomes for LFF and HFF from baseline to the end of follow‐up. Greater improvements in glycemic control, as measured by percentage changes of HbA1c and FBG, were achieved in patients with both LFF (−9.67% ± 20.29% in HbA1c and −2.21% ± 43.08% in FBG, both *P* < .0001) and HFF (−12.14% ± 19.78% in HbA1c and −4.95% ± 37.96% in FBG, both *P* < .0001) (Table 2 and Table S1). Moreover, other metabolic parameters were improved in both groups after follow‐up, except for fasting C‐peptide. However, reduction in BMI was only observed in the HFF group (−0.53% ± 6.59%, *P* < .0001 in HFF and 0.12% ± 6.64% in LFF, *P* > .05; Table 2 and Table S1). To investigate the effectiveness of different follow‐up frequencies on metabolic parameters, the between‐group percentage changes of metabolic parameters were analyzed. Compared to LFF, HFF was associated with a statistically significant reduction in FBG, HbA1c, BMI, waist circumference, SBP, DBP, total cholesterol, and low‐density lipoprotein (LDL) cholesterol after full adjustments for the confounders (Table 2). Because there was a significant difference between the two groups (Table 1), we further conducted propensity score matching (PSM) with a logistic model that included age, sex, education level, and duration of follow‐up, BMI, SBP, HbA1c, total cholesterol, and duration of diabetes. After PSM, participants in the LFF and HFF groups were well balanced (Table S2). In total, 3000 LFF and 6000 HFF patients were compared. We found that metabolic parameters, including FBG, DBP, HbA1c, triglyceride, total cholesterol, and LDL cholesterol, were much more improved in HFF, suggesting that the results were mostly equivalent (Table S3).

**TABLE 2:** Clinical outcomes in T2DM patients after LFF and HFF

	Total		LFF		HFF		
	Follow‐up	Percentage change (%)	Follow‐up	Percentage change (%)	Follow‐up	Percentage change (%)	*P* value
Fasting glucose (mmol/L)	8.22 ± 3.01	−3.58 ± 40.62	8.48 ± 3.31	−2.21 ± 43.08	7.97 ± 2.65	−4.95 ± 37.96	<.0001
Fasting C‐peptide (μg/L)	2.30 ± 1.32	19.39 ± 248.13	2.32 ± 1.34	19.65 ± 316.91	2.29 ± 1.30	19.12 ± 140.39	.89
BMI (kg/m^2^)	25.8 ± 3.6	−0.19 ± 6.62	25.8 ± 3.6	0.12 ± 6.64	25.8 ± 3.6	−0.53 ± 6.59	<.0001
Visceral fat area (cm^2^)	96.2 ± 39.1	3.20 ± 74.12	95.2 ± 39.4	3.86 ± 76.15	97.3 ± 38.7	2.50 ± 71.92	.49
Waist circumference (cm)	91.1 ± 9.5	−0.22 ± 6.51	90.8 ± 9.4	−0.10 ± 6.26	91.4 ± 9.6	−0.36 ± 6.82	.022
SBP (mm Hg)	131.3 ± 17.6	0.48 ± 14.60	131.9 ± 17.7	0.79 ± 14.49	130.7 ± 17.6	0.13 ± 14.71	.0004
DBP (mm Hg)	76.5 ± 10.5	0.09 ± 14.69	77.0 ± 10.2	0.44 ± 14.40	75.9 ± 10.8	−0.28 ± 14.98	.0006
HbA1c (%)	7.38 ± 1.53	−10.91 ± 20.07	7.64 ± 1.67	−9.67 ± 20.29	7.12 ± 1.34	−12.14 ± 19.78	<.0001
Triglyceride (mmol/L)	1.9 ± 1.9	5.06 ± 79.54	2.0 ± 2.0	6.32 ± 79.01	1.9 ± 1.8	3.79 ± 80.05	.025
Total cholesterol (mmol/L)	4.6 ± 1.2	−2.01 ± 25.91	4.7 ± 1.2	−1.62 ± 25.60	4.6 ± 1.2	−2.39 ± 26.21	.0048
HDL cholesterol (mmol/L)	1.3 ± 0.4	9.64 ± 58.85	1.3 ± 0.4	10.69 ± 73.79	1.3 ± 0.4	8.58 ± 38.53	.0094
LDL cholesterol (mmol/L)	2.6 ± 0.9	−4.54 ± 41.86	2.7 ± 1.0	−4.01 ± 44.91	2.6 ± 0.9	−5.07 ± 38.56	.037

*Note*: Metabolic parameters within groups are shown as mean ± SD. *P* values for the between‐group percentage changes were evaluated using multivariable linear regression models, adjusted for age, sex, education level, duration of follow‐up, BMI, SBP, HbA1c, total cholesterol, and duration of diabetes.

Abbreviations: BMI, body mass index; DBP, diastolic blood pressure; HbA1c, glycosylated hemoglobin; HDL, high‐density lipoprotein; HFF, higher‐frequency follow‐up; LDL, low‐density lipoprotein; LFF, lower‐frequency follow‐up; SBP, systolic blood pressure; T2DM, type 2 diabetes mellitus.

### 3.4 Effect of follow‐up frequency on HbA1c change in subgroups

**FIGURE 3:**
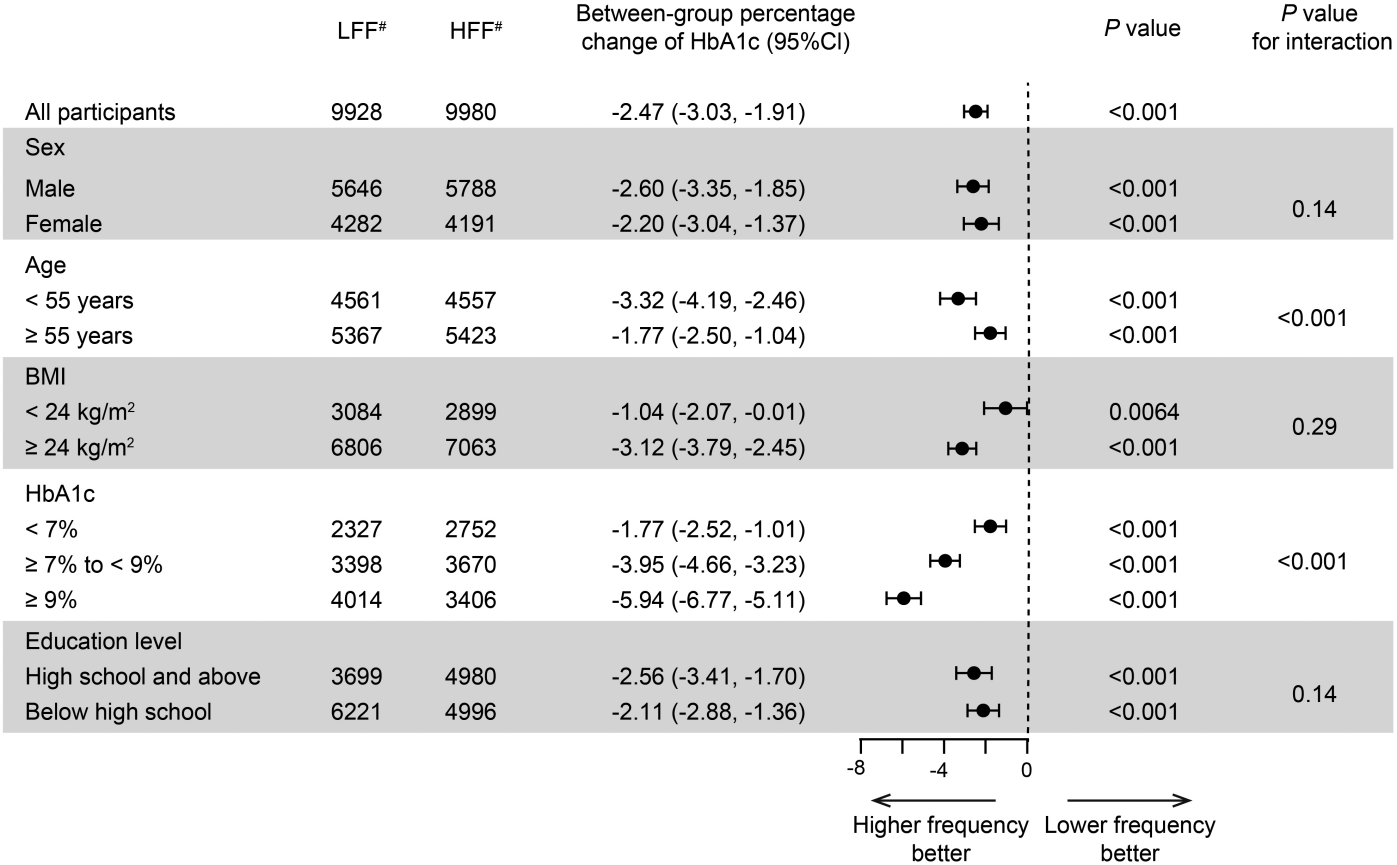
Subgroup analyses of association of the between‐group percentage changes of HbA1c with the follow‐up frequency. Adjusted for age, sex, education level, and duration of follow‐up, BMI, SBP, HbA1c, total cholesterol and duration of diabetes, if not be stratified. BMI body mass index, HbA1c hemoglobin A1c, SBP systolic blood pressure. #Numbers do not always sum to group totals due to missing information for some subgroup variables

The relationship between follow‐up frequency and HbA1c was further investigated with stratified analysis in five subgroups that were defined according to the baseline characteristics (Figure 3). These subgroup analyses showed that the between‐group percentage change of HbA1c was significant in all HbA1c level groups of <7%, 7% to <9%, and ≥9% after full adjustments for the confounders. The magnitude of HbA1c reduction increased with the rise in baseline HbA1c (*P* for interaction <.001, Figure 3). The HbA1c percentage changes in the subgroups with baseline HbA1c < 7%, 7% to ≤9%, and >9% were 1.77%, 3.95%, and 5.94%, respectively (Figure 3). Analyses stratified by age (<55 and ≥55 years) also showed that there were significant differences in percentage change of HbA1c between LFF and HFF (*P* for interaction <.001, Figure 3). There were no significant interactions among the subgroups of sex, BMI, and education level after adjusting for major covariables (*P* for interaction >.05, Figure 3).

## 4 DISCUSSION

Diabetes is becoming a global public health crisis, affecting578 million people worldwide by 2030 and 700 million by 2045^19^ and imposing a substantial cost burden on the Chinese health care system.^20^ The MMCs were launched nationwide in China to provide a new metabolic disease management model with the objective to improve adherence to and the effectiveness of treatment.^5^ In order to achieve cost‐effectiveness under the conditions of high prevalence of T2DM in various clinical settings,^21^ it is critical to understand the appropriate follow‐up model, including visit frequency, to optimize the efficiency of diabetes management.^15^ In this prospective, multicenter, real‐world study of nearly 20 000 participants with T2DM, our data demonstrated that compared to those with LFF, individuals with HFF (more than two follow‐up visits per year) had significant improvements in a variety of metabolic parameters after follow‐up, regardless of diabetic control status at baseline. Similar to our results, Asao et al^15^ found that regardless of diabetic control, the outcome in diabetic patients was an improvement in HbA1c, which was associated with an intensive frequency of follow‐up. Of particular interest, subgroup analysis indicated that compared with those in LFF, participants in HFF with younger age (age < 55 years) or high HbA1c level (HbA1c > 7%) at baseline had significantly enhanced benefits. These results indicate the importance of conducting hierarchical management regarding individual patients' characteristics at baseline, which could increase the effectiveness and reduce the public health burden in long‐term management of T2DM patients in China. Specifically, in this study, we recommend that intensive follow‐up may be more beneficial for glycemic control in patients with younger age and higher HbA1c at baseline.

In the baseline analysis, there were a number of factors that were associated with follow‐up frequency. We suggested that education level may affect the behavioral pattern of follow‐up—patients with higher education level (high school and above) may have better treatment compliance, resulting in better metabolic outcomes. Recently, our group revealed that less education (lower than high school) is one of the socioeconomic risk factors which contribute to the diabetes risk in adults.^22^ This work with real‐world, large sample data extended previous research on the association between follow‐up frequency and glycemic control, and we evaluated more additional metabolic parameters. Our results demonstrate that HFF was associated with greater improvements in metabolic outcomes, including FBG, HbA1c, blood pressure, and cholesterol level in HFF participants. For well‐controlled diabetes, the benefits of HFF were controversial.^13^, ^15^, ^23^ A previous randomized controlled trial (3‐month and 6‐month follow‐up for 18 months) and a retrospective cohort study (monthly and bimonthly follow‐up for 12 months) showed that frequent follow‐up did not affect blood glucose control.^13^, ^23^ Our results confirmed the findings that in the subgroup with baseline HbA1c < 7%, the between‐group percentage change of HbA1c was statistically different, but the clinical benefit was limited and below the average level (−1.77%; 95% CI −2.52%, −1.01%; *P* < .001; Figure 3). Morrison et al^12^ reported that patients with diabetes and elevated HbA1c, BP, and/or LDL cholesterol achieved well‐controlled targets with high follow‐up frequency. In our cohort, the between‐group percentage change of HbA1c in the subgroups of HbA1c < 7%, 7% to ≤9%, and >9% was continuously increased, suggesting that participants with higher baseline HbA1c levels will benefit more from HFF. Besides, our work identified that participants in subgroups with younger age (<55 years) at baseline achieved better glycemic control through HFF. There were no significant differences for interactions between education level, BMI, and follow‐up frequency. We supposed that the MMCs provided diabetes self‐management support to the T2DM patients and diminished the disadvantage and risk of low education levels for blood glucose control. Moreover, for subgroups analysis of BMI, no interaction was detected in the stratified analysis after adjustment. There was significant interaction for BMI and between‐group percentage change of HbA1c without adjusting for HbA1c (*P* for interaction = .022), suggesting that difference in BMI stratification might be dependent on the HbA1c level at baseline.

The increasing cost of diabetes care has put a heavy economic burden on society.^24^ In our study, we found that well‐controlled individuals (HbA1c < 7%) with HFF in MMCs improved limited metabolic outcomes compared to LFF, providing valuable information for health care management and policies to reduce unnecessary expenses and make diabetes care more cost‐effective. Expanding the scheduled follow‐up interval may also perceive the MMCs as having several benefits for patients. It encourages adherence to treatment and provides more convenient access to diabetes management. Moreover, HFF is critical and essential for the indicated subgroups of T2DM, as described above. For uncontrolled T2DM, appropriate follow‐up frequency is a key factor to engage in diabetes care. Programs in MMCs to targeted T2DM patients with different baseline characteristics are necessary to be designed for improving efficiency of follow‐up.

There are several limitations to these findings. First, the median frequency is 2.7 times per year for HFF. Since participants with more than four follow‐up visits per year represent only a small population (Figure 2), we did not provide evidence whether more frequent follow‐up visits (eg, five times per year or more) are likely to further improve blood glucose control. Second, even though we adjusted for many potential confounders, other residual confounding, such as diet, family income, and access to internet‐based self‐management support provided by the app and social software platform of the MMCs may also influence the change of metabolic outcomes. Third, we did not evaluate the association between follow‐up frequency and diabetes‐related complications such as cardiovascular events. Therefore, more evidence from long‐term follow‐up in these participants is needed in the future.

In conclusion, the MMCs are an innovative system and efficient strategy to manage metabolic diseases in China. After an average of 20‐month follow‐up, T2DM patients achieved significant improvements in metabolic outcomes. HHF for more than two times per year is suggested to be a potentially beneficial way for glycemic control especially in participants with younger age (<55 years) and worse HbA1c (>7%) at baseline.

## DISCLOSURE

The authors have nothing to disclose.

## ETHICAL STATEMENT

This study was approved by the institutional review board of Ruijin Hospital affiliated to Shanghai Jiao Tong University School of Medicine, the leading MMC center, and other participating centers, if necessary. This study was performed in accordance with the Declaration of Helsinki, and all study participants provided written informed consent.

## Supporting information


Appendix S1
