# Supplementary material for: Follow‐up frequency and clinical outcomes in patients with type 2 diabetes: A prospective analysis based on multicenter real‐world data
Source: J Diabetes. 2022 May 25;14(5):306–14. doi: 10.1111/1753-0407.13271 (PMC9366569; doi:10.1111/1753-0407.13271)
Supplement: Supplementary file 1 — Appendix S1 [file JDB-14-306-s001.docx]

|  | **LFF (n=9,928)** | | **HFF (n=9,980)** | |
| --- | --- | --- | --- | --- |
|  | Change from baseline | *P* value | Change from baseline | *P* value |
| Fasting glucose (mmol/L) | -1.07 ± 4.29 | <0.0001 | -1.20 ± 3.78 | <0.0001 |
| Fasting C-peptide (μg/L) | 0.02 ± 1.16 | 0.173 | 0.02 ± 1.14 | 0.145 |
| BMI (kg/m^2^) | -0.02 ± 1.68 | 0.212 | -0.21 ± 1.71 | <0.0001 |
| Visceral fat (m^2^) | -5.3 ± 29.7 | <0.0001 | -5.7 ± 28.8 | <0.0001 |
| Waist circumference (cm) | -0.30 ± 5.38 | <0.0001 | -0.57 ± 6.00 | <0.0001 |
| SBP (mmHg) | -0.48 ± 18.89 | 0.016 | -1.26 ± 19.23 | <0.0001 |
| DBP (mmHg) | -0.61 ± 10.90 | <0.0001 | -1.11 ± 11.30 | <0.0001 |
| HbA1c (%) | -1.11 ± 2.11 | <0.0001 | -1.31 ± 2.07 | <0.0001 |
| Triglyceride (mmol/L) | -0.27 ± 2.23 | <0.0001 | -0.32 ± 2.23 | <0.0001 |
| Total cholesterol (mmol/L) | -0.23 ± 1.26 | <0.0001 | -0.30 ± 1.33 | <0.0001 |
| HDL cholesterol (mmol/L) | 0.07 ± 0.39 | <0.0001 | 0.06 ± 0.33 | <0.0001 |
| LDL cholesterol (mmol/L) | -0.29 ± 0.97 | <0.0001 | -0.33 ± 1.02 | <0.0001 |
| HbA1c < 7%, n (%) | 1,902 (28.71%) | <0.0001 | 2,774 (27.37%) | <0.0001 |

**Supplementary Table 1-Changes in metabolic parameters in LFF and HFF groups after follow-up**

Changes in metabolic parameters within groups are shown as mean ± SD. *P* values are for the baseline and follow-up comparison in LFF and HFF groups via the paired sample *t*-test for continuous outcomes and chi-square test for HbA1c < 7%.

LFF lower frequency follow-up, HFF higher frequency follow-up, BMI body mass index, SBP systolic blood pressure, DBP diastolic blood pressure, HbA1c glycated hemoglobin, LDL low-density lipoprotein, HDL high-density lipoprotein.

**Supplementary Table 2-Characteristics of the propensity score matched pairs in LFF and HFF**

|  | Total | LFF | HFF | P value |
| --- | --- | --- | --- | --- |
| N | 9,000 | 3,000 | 6,000 |  |
| Age (years) | 55.3 ± 10.8 | 55.3 ± 10.4 | 55.3 ± 10.9 | 0.92 |
| Male, n (%) | 4,943 (54.9%) | 1,673 (55.8%) | 3,270 (54.5%) | 0.27 |
| Duration of diabetes (years) | 5.9 (1.4, 12.0) | 6.2 (1.8, 12.3) | 5.8 (1.3, 11.9) | 0.071 |
| History of hypertension | 4,004 (44.5%) | 1,337 (44.6%) | 2,667 (44.5%) | 0.93 |
| Education level-High school and above, n (%) | 3,091 (34.3%) | 1,012 (33.7%) | 2,079 (34.7%) | 0.40 |
| Ideal smoking, n (%) | 6,839 (76.3%) | 2,271 (76.0%) | 4,568 (76.4%) | 0.68 |
| Drinking, n (%) | 942 (10.5%) | 316 (10.6%) | 626 (10.5%) | 0.91 |
| Fasting glucose (mmol/L) | 9.43 ± 3.65 | 9.46 ± 3.81 | 9.41 ± 3.57 | 0.52 |
| Fasting C-peptide (μg/L) | 2.07 (1.41, 2.87) | 2.10 (1.43, 2.94) | 2.05 (1.41, 2.81) | 0.024 |
| BMI (kg/m^2^) | 25.87 ± 3.61 | 25.85 ± 3.62 | 25.87 ± 3.61 | 0.80 |
| Visceral fat area (cm^2^) | 99.8 ± 39.8 | 99.4 ± 40.5 | 100.0 ± 39.4 | 0.52 |
| Waist circumference (cm) | 91.2 ± 9.7 | 91.2 ± 9.9 | 91.3 ± 9.6 | 0.67 |
| SBP (mmHg) | 133.0 ± 18.7 | 133.1 ± 18.9 | 133.0 ± 18.6 | 0.95 |
| DBP (mmHg) | 77.6 ± 11.6 | 77.7 ± 11.4 | 77.5 ± 11.7 | 0.63 |
| HbA1c (%) | 8.62 ± 2.08 | 8.67 ± 2.12 | 8.59 ± 2.07 | 0.085 |
| Triglyceride (mmol/L) | 1.59 (1.09, 2.40) | 1.60 (1.11, 2.42) | 1.58 (1.09, 2.39) | 0.43 |
| Total cholesterol (mmol/L) | 4.92 ± 1.31 | 4.93 ± 1.29 | 4.91 ± 1.32 | 0.57 |
| HDL cholesterol (mmol/L) | 1.21 ± 0.34 | 1.21 ± 0.34 | 1.21 ± 0.35 | 0.92 |
| LDL cholesterol (mmol/L) | 2.96 ± 1.02 | 2.98 ± 1.01 | 2.94 ± 1.03 | 0.13 |
| Duration of follow-up (months) | 19.2 ± 8.9 | 19.0 ± 8.6 | 19.3 ± 9.00 | 0.080 |
| HbA1c < 7%, n (%) | 2,251 (25.0%) | 736 (24.5%) | 1,515 (25.3%) | 0.48 |

Data are presented as mean ± SD, median (25%,75%), or n (%). The groups were compared via ANOVA test for the continuous outcomes and χ2-test for dichotomous variable (sex, education level, history of hypertension, ideal smoking, drinking, HbA1c ≤ 7%).

LFF lower frequency follow-up, HFF higher frequency follow-up, BMI body mass index, SBP systolic blood pressure, DBP diastolic blood pressure, HbA1c glycated hemoglobin, LDL low-density lipoprotein, HDL high-density lipoprotein

**Supplementary Table 3-Clinical outcomes in T2DM patients of LFF and HFF after propensity score matching**

|  | Total | | LFF | | HFF | |  |
| --- | --- | --- | --- | --- | --- | --- | --- |
|  | Follow-up | Percentage  change (%） | Follow-up | Percentage  change (%） | Follow-up | Percentage  change (%） | *P* value |
| Fasting glucose (mmol/L) | 8.22 ± 2.99 | -4.63 ± 39.56 | 8.49 ± 3.38 | -1.82 ± 42.70 | 8.09 ± 2.76 | -6.04 ± 37.81 | <0.0001 |
| Fasting C-peptide (μg/L) | 2.31 ± 1.31 | 17.16 ± 138.44 | 2.33 ± 1.29 | 14.55 ± 127.77 | 2.30 ± 1.32 | 18.56 ± 143.81 | 0.28 |
| BMI (kg/m^2^) | 25.7 ± 3.5 | -0.16 ± 6.77 | 25.7 ± 3.6 | -0.03 ± 6.98 | 25.7 ± 3.5 | -0.24 ± 6.65 | 0.15 |
| Visceral fat area (cm^2^) | 95.3 ± 38.1 | 3.68 ± 81.80 | 95.2 ± 37.6 | 3.84 ± 90.35 | 95.3 ± 38.3 | 3.59 ± 77.08 | 0.94 |
| Waist circumference (cm) | 90.8 ± 9.4 | -0.10 ± 6.82 | 90.6 ± 9.3 | -0.18 ± 6.56 | 90.9 ± 9.5 | -0.04 ± 6.98 | 0.42 |
| SBP (mmHg) | 131.8 ± 17.9 | 0.16 ± 14.75 | 132.2 ± 17.5 | 0.56 ± 14.43 | 131.5 ± 18.1 | -0.05 ± 14.92 | 0.064 |
| DBP (mmHg) | 76.3 ± 10.6 | -0.30 ± 14.69 | 76.8 ± 10.1 | 0.31 ± 14.25 | 76.0 ± 10.9 | -0.63 ± 14.91 | 0.0024 |
| HbA1c (%) | 7.35 ± 1.50 | -11.52 ± 20.03 | 7.63 ± 1.69 | -9.16 ± 19.82 | 7.21 ± 1.37 | -12.70 ± 20.03 | <0.0001 |
| Triglyceride (mmol/L) | 1.9 ± 1.9 | 5.45 ± 87.12 | 2.0 ± 1.9 | 9.09 ± 86.89 | 1.8 ± 1.8 | 3.61 ± 87.19 | 0.0043 |
| Total cholesterol (mmol/L) | 4.6 ± 1.2 | -2.65 ± 26.34 | 4.7 ± 1.2 | -1.34 ± 26.32 | 4.6 ± 1.2 | -3.30 ± 26.32 | 0.00054 |
| HDL cholesterol (mmol/L) | 1.3 ± 0.4 | 8.69 ± 45.30 | 1.3 ± 0.4 | 9.65 ± 50.74 | 1.3 ± 0.4 | 8.20 ± 42.29 | 0.13 |
| LDL cholesterol (mmol/L) | 2.6 ± 0.9 | -4.66 ± 43.81 | 2.7 ± 1.0 | -3.41 ± 51.08 | 2.6 ± 0.9 | -5.28 ± 39.64 | 0.035 |

Metabolic parameters within groups are shown as mean ± SD. *P* values for the between-group percentage changes were evaluated using multivariable linear regression models, adjusted for age, sex, education level, and duration of follow-up, BMI, SBP, HbA1c, total cholesterol and duration of diabetes.

LFF lower frequency follow-up, HFF higher frequency follow-up, BMI body mass index, SBP systolic blood pressure, DBP diastolic blood pressure, HbA1c glycated hemoglobin, LDL low-density lipoprotein, HDL high-density lipoprotein.
